# A high-carbohydrate diet induces greater inflammation than a high-fat diet in mouse skeletal muscle

**DOI:** 10.1590/1414-431X20199039

**Published:** 2020-02-14

**Authors:** M.M. Antunes, G. Godoy, C.B. de Almeida-Souza, B.A. da Rocha, L.G. da Silva-Santi, L.N. Masi, F. Carbonera, J.V. Visentainer, R. Curi, R.B. Bazotte

**Affiliations:** 1 Departamento de Farmacologia e Terapêutica, Universidade Estadual de Maringá, Maringá, PR, Brasil; 2 Programa de Pós-Graduação Interdisciplinar em Ciências da Saúde, Universidade Cruzeiro do Sul, São Paulo, SP, Brasil; 3 Departmento de Química, Universidade Estadual de Maringá, Maringá, PR, Brasil

**Keywords:** Saturated fatty acids, Monounsaturated fatty acids, Polyunsaturated fatty acids, n-6/n-3 PUFA ratio, SFA/n-3 PUFA ratio

## Abstract

We previously reported that both the high-carbohydrate diet (HCD) and high-fat diet (HFD) given for two months promote lipid deposition and inflammation in the liver and brain of mice. The results obtained indicate a tissue-specific response to both diets. Herein, we compared the effects of HCD and HFD on fatty acid (FA) composition and inflammation in the gastrocnemius muscle. Male Swiss mice were fed with HCD or HFD for 1 or 2 months. Saturated FA (SFA), monounsaturated FA (MUFA), n-3 polyunsaturated FA (n-3 PUFA), and n-6 PUFA were quantified. The activities of stearoyl-CoA desaturase 1 (SCD-1), Δ-6 desaturase (D6D), elongase 6, and *de novo* lipogenesis (DNL) were estimated. As for indicators of the inflammatory tissue state, we measured myeloperoxidase (MPO) activity and gene expression of F4/80, tumor necrosis factor-α (TNF-α), interleukin (IL)-4, IL-6, and IL-10. The HCD led to a lower deposition of SFA, MUFA, n-3 PUFA, and n-6 PUFA compared to HFD. However, the HCD increased arachidonic acid levels, SFA/n-3 PUFA ratio, DNL, SCD-1, D6D, and MPO activities, and expression of IL-6, contrasting with the general idea that increased lipid deposition is associated with more intense inflammation. The HCD was more potent to induce skeletal muscle inflammation than the HFD, regardless of the lower lipid accumulation.

## Introduction

Both blood fatty acids (FA) and tissue-triacylglycerol-derived FA are sources of ATP for skeletal muscle contraction (1). Lipids stored in skeletal muscles play an important role as an energy supply during physical exercise. However, abnormal lipid deposition in the skeletal muscles of sedentary and obese individuals is associated with inflammation, insulin resistance, type 2 diabetes, cardiovascular diseases, and myopathies (2,3).

Diet-induced obesity promotes insulin resistance (4,5), lipid accumulation (6,7), and inflammation (8,9) in skeletal muscle. The regulation of skeletal muscle FA composition is not fully understood; however, it markedly changes with dietary macronutrient composition. Skeletal muscle FA composition varies according to the proportion of the FA present in the diet (10,11). These studies, however, do not differentiate the effects of macronutrient composition from those caused by obesity.

As described in other reports (8,12,13), we previously demonstrated that a high-carbohydrate diet (HCD) given for 2 months leads to a similar body weight gain compared with a high-fat diet (HFD) in Swiss mice (14,15), allowing the evaluation of diet-induced changes in FA composition and inflammatory markers without the influence of obesity. In these studies, we reported changes in FA composition and inflammatory markers in the liver (14) and brain (15). Herein, we extended the previous studies to skeletal (gastrocnemius) muscle of male Swiss mice.

## Material and Methods

### Animals and experimental design

The Scientific Advisory Committee on Animal Care of the State University of Maringá (protocol No. 3105210717) approved the experimental procedures of the present study, following the International Guidelines for the Use and Care of Laboratory Animals.

**Figure 1:**
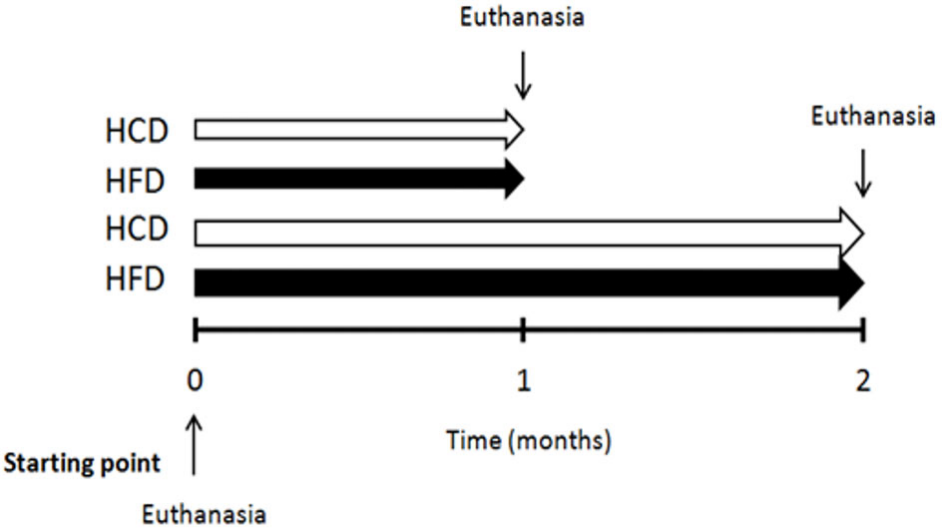
Experimental design. All mice received standard rodent chow before starting (time 0) and a high-fat diet (HFD) or a high-carbohydrate diet (HCD) was administered for 1 or 2 months.

Weaned mice received standard rodent chow (Nuvilab™, Brazil) (53.3% carbohydrates, 22% proteins, and 4.5% lipids). The starting point (Time 0) was considered to be when the mice reached six weeks of age (about 33 g body weight). They were then randomly divided into groups that received HCD (73.8% carbohydrates, 14.2% proteins, and 4% lipids) or HFD (36.5% carbohydrates, 20.3% proteins, and 35.2% lipids) for 1 or 2 months (Figure 1). Specific nutrients and FA composition of the diets (HCD or HFD) are described in our previous publications (14,16). All mice had free access to food and water. We recorded caloric intake and body weight throughout the study.

Overnight-fasted mice (from 17:00 to 08:00), as described in our previous studies (9,14–17), were then euthanized by decapitation. Gastrocnemius muscle from both legs was removed, frozen in liquid nitrogen, and stored at –80°C until analysis.

### Fatty acids composition

We used the method of Bligh and Dyer (18) on a reduced-scale to extract total lipids from the gastrocnemius muscle. FA methyl esters (FAME) were prepared by ultrasound-assisted total lipid methylation, as described by Santos et al. (19). FAME was separated by gas chromatography. Retention times and peak areas were determined using the Chrom-Quest™ software (Thermo Scientific™, USA). FA contents in the muscles were reported as mg/g of total fat.

### Estimated *de novo* lipogenesis (DNL) and activities of stearoyl-CoA desaturase 1 (SCD-1), Δ-6 desaturase (D6D), and elongase 6

DNL and activities of SCD-1, D6D, and elongase 6 were estimated using the product/precursor ratios of individual FA in lipid esters as follows: DNL as the ratio of 16:0/18:2n-6; SCD-1 activity index as the ratio of 16:1n-7/16:0; D6D activity index as the ratio of 18:3n-6/18:2n-6; and elongase 6 activity index as the ratio of 18:0/16:0.

### Determination of myeloperoxidase (MPO) activity

Gastrocnemius muscles of mice fed with HCD or HFD for 2 months were homogenized in phosphate-buffered saline (PBS), and the homogenate was stirred in a vortex and centrifuged (500 *g*, 4°C) for 5 min. The activity of MPO was measured in tissue supernatants (10 µL) in triplicate. PBS (0.2 mL) containing o-dianisidine dihydrochloride (4.2 mg), double-distilled water (22.5 mL), potassium phosphate buffer (2.5 mL, pH=6), and H_2_O_2_ (10 µL, 1%) was also added. The enzyme reaction was stopped by the addition of 30 µL sodium acetate (2.23 g in 20 mL of double-distilled water). MPO activity was determined at 460 nm, using a microplate spectrophotometer (Asys Expert Plus, Biochrom, UK), and is reported as absorbance (Ab).

### Gene expression measurement

F4/80, tumor necrosis factor-α (TNF-α), interleukin (IL)-6, IL-4, and IL-10 mRNA expressions were measured in the gastrocnemius muscle of mice fed with HCD or HFD for 2 months. The gastrocnemius muscles (20 mg) were powdered in liquid nitrogen and total RNA was extracted using Trizol reagent (Invitrogen Life Technologies, USA). Reverse transcription to cDNA was performed using the High-Capacity cDNA kit (Applied Biosystems, USA). Gene expression was evaluated by real-time PCR using SYBR Green as the fluorescent dye (Invitrogen Life Technologies).

The quantification of gene expression was performed using the comparative Ct method (Ct: threshold cycle, the cycle number in which the PCR product reaches the detection threshold). β2-microglobulin gene (β2m) expression was used as a reference.

The primer sequences were: F4/80, NM_010130.4, sense CCTGAACATGCAACCTGCCAC, antisense GGGCATGAGCAGBCTGTAGGATC; TNF-α, NM_001278601.1, sense TCTTCTCATTCCTGCTTGTGGC, antisense CACTTGGTGGTTTGCTACGACG; IL-6, NM_001314054.1, sense GGTAGCATCCATCATTTCTTTG, antisense CGGAGAGGAGACTTCACAAGAG; IL-4, NM_021283.2, sense CCATATCCACGGATGCGACA, antisense CTGTGGTGTTCTTCGTTGCTG; IL-10, NM_010548.2, sense TGCCAAGCCTTATCGGAAATG, antisense AAATCGATGACAGCGCCTCAG.

### Statistical analysis

The results are reported as means±SE. One-way ANOVA followed by the Tukey’s *post*-test was used to evaluate differences between 0, 1, and 2 months. The Student's *t*-test was used to assess differences between the HCD and HFD groups. Statistical analyses were performed using GraphPad Prism 5.0 software (USA). A P-value <0.05 indicated statistical significance.

## Results

### Caloric intake, body weight, and gastrocnemius muscle weights

The daily caloric intake was 32.6±5.8 kcal/day for HCD group and 22.6±2.0 kcal/day for HFD group after 1 month of diet intervention, and 29.4±2.0 kcal/day for HCD group and 20.9±0.5 kcal/day for HFD group after 2 months.

The final body weight was 45.8±1.4 g for HCD group and 45.0±2.2 g for HFD group after 1 month, and 51.0±1.9 g for HCD group and 50.6±1.5 g for HFD group after 2 months.

The gastrocnemius muscle weights were 0.47±0.01 g in the HCD group and 0.45±0.01 g in the HFD group, 2 months after starting the diets.

### FA composition, estimated DNL, and activities of SCD-1, D6D, and elongase 6

**Figure 2:**
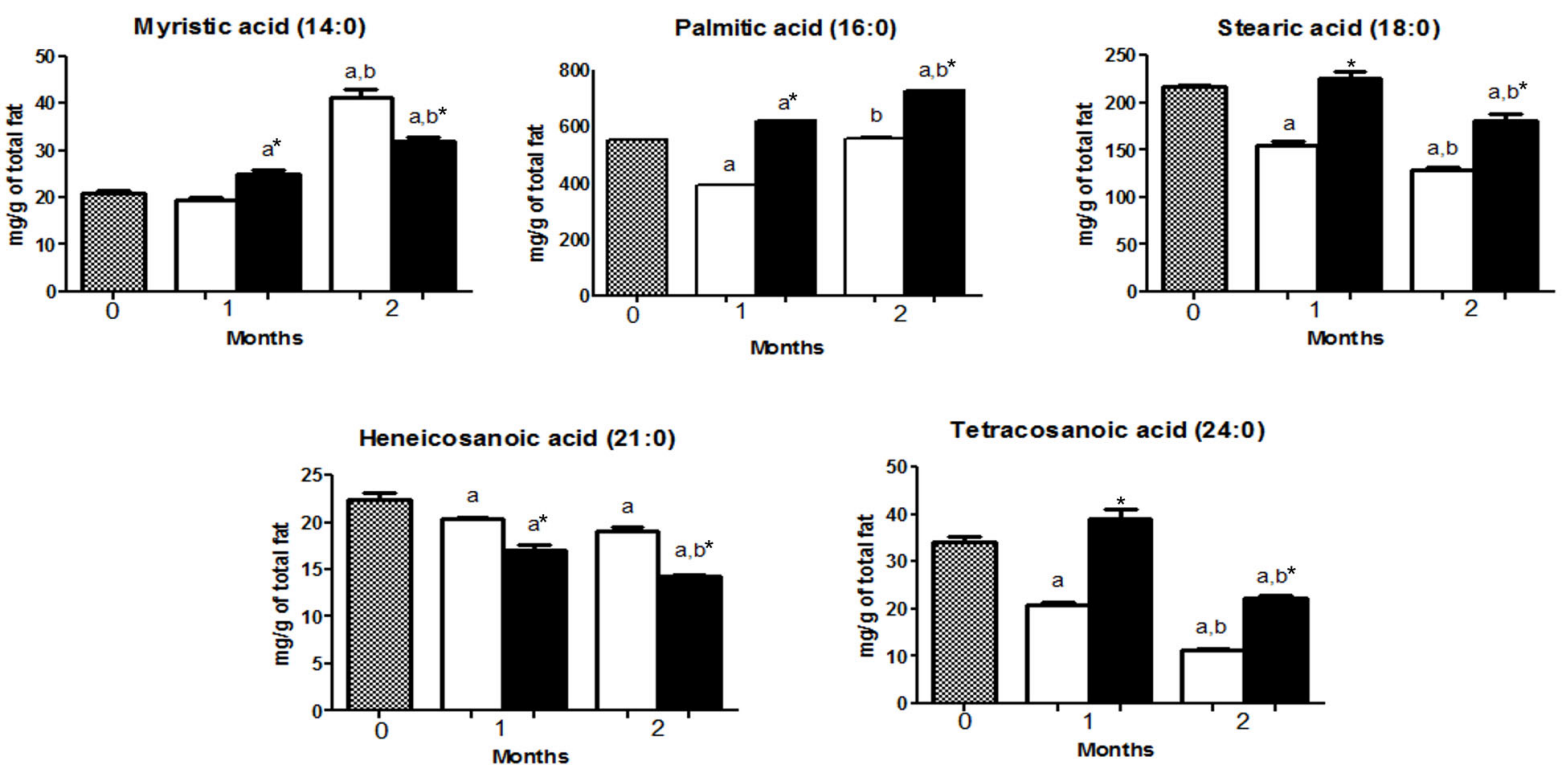
Saturated fatty acid (SFA) composition in the gastrocnemius muscle of mice at 0 (before starting the diets, gray bars) or fed with a high-carbohydrate diet (HCD, white bars) or high-fat diet (HFD, black bars) for 1 or 2 months. The concentrations of SFA are reported as means±SE. *P<0.05 compared to HCD group (Student's *t*-test). ^a^P<0.05 compared to time 0; ^b^P<0.05 compared to 1 month (one-way ANOVA).

**Figure 3 f03:**
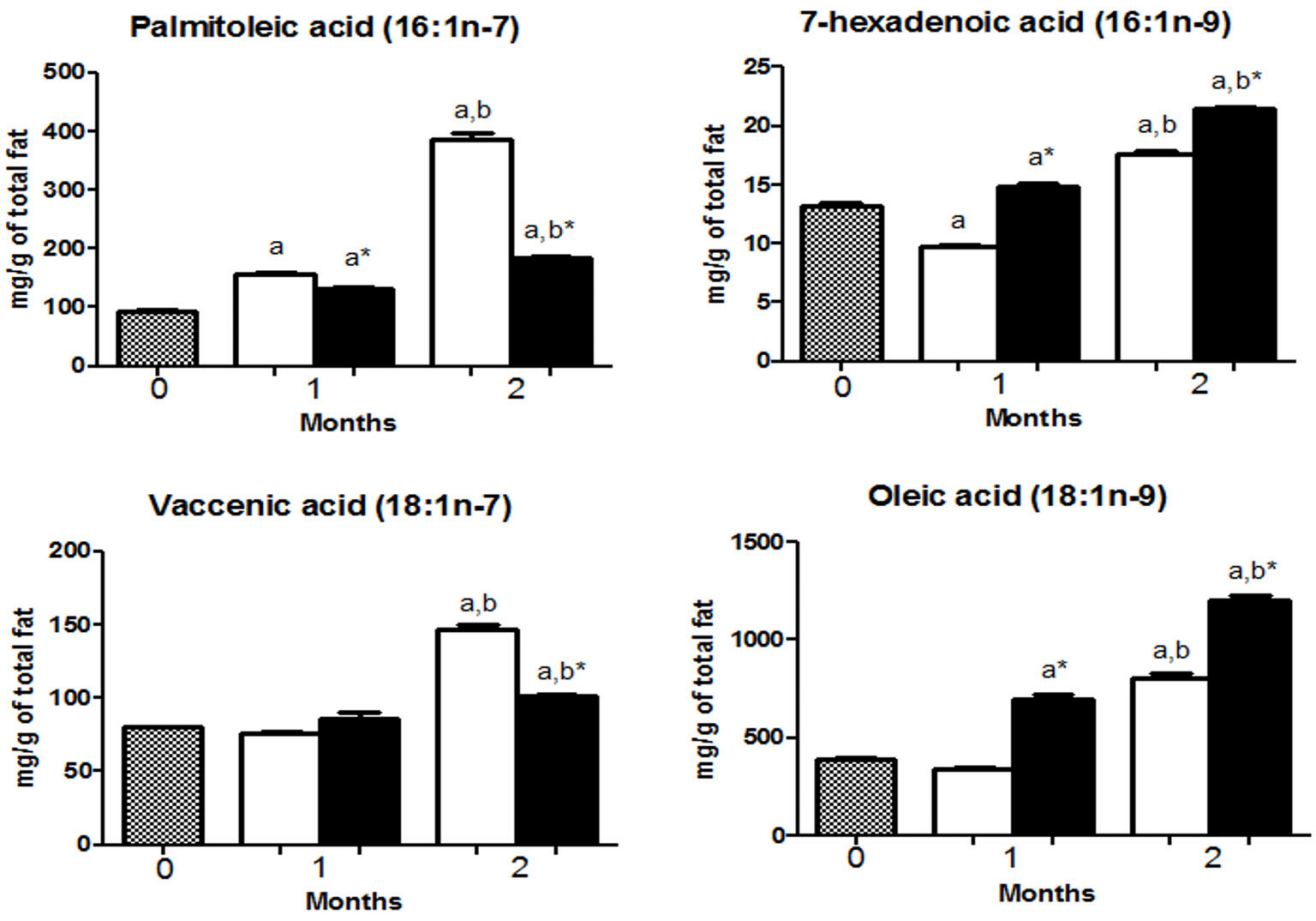
Monounsaturated fatty acids (MUFAs) composition in the gastrocnemius muscle of mice at 0 (before starting the diets, gray bars) or fed with a high-carbohydrate diet (HCD, white bars) or high-fat diet (HFD, black bars) for 1 or 2 months. The concentrations of MUFAs are reported as means±SE. *P<0.05 compared to HCD group (Student's *t*-test). ^a^P<0.05 compared to time 0; ^b^P<0.05 compared to 1 month (one-way ANOVA).

**Figure 4 f04:**
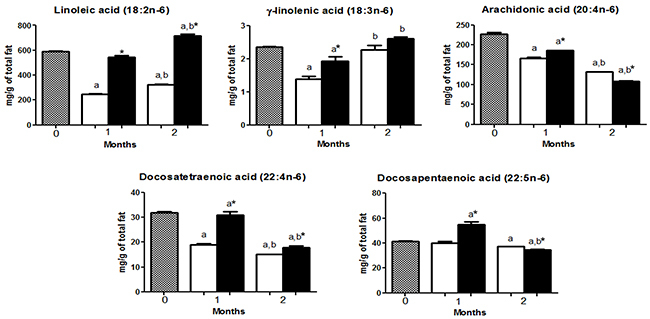
Polyunsaturated n-6 fatty acid (n-6 PUFA) composition in the gastrocnemius muscle of mice at 0 (before starting the diets, gray bars) or fed with a high-carbohydrate diet (HCD, white bars) or high-fat diet (HFD, black bars) for 1 or 2 months. The levels of n-6 PUFA are reported as means±SE. *P<0.05 compared to HCD group (Student's *t*-test). ^a^P<0.05 compared to time 0; ^b^P<0.05 compared to 1 month (one-way ANOVA).

HCD and HFD mice had a higher content of palmitic acid (16:0), oleic acid (18:1 n-9), and linoleic acid (18:2n-6) compared with other FA (Figures 2, 3, and 4).

Heneicosanoic acid (21:0) and palmitoleic acid (16:1n-7) were increased (P<0.05) in the HCD group compared to HFD group, after 1 and 2 months. Myristic acid (14:0), vaccenic acid (18:1n-7), arachidonic acid (20:4n-6), docosapentaenoic acid (22:5n-6), and eicosapentaenoic acid (20:5n-3) were higher (P<0.05) in the HFD only after 1 month of diet intervention but did not differ (HFD *vs* HCD) after 2 months (Figures 2, 3, and 4).

Palmitic acid (16:0), stearic acid (18:0), tetracosanoic acid (24:0), 7-hexadecanoic acid (16:1n-9), oleic acid (18:1n-9), linoleic acid (18:2n-6), docosatetraenoic acid (22:4n-6), α-linolenic acid (18:3n-3), and docosahexaenoic acid (22:6n-3) were higher (P<0.05) in the HFD group compared to HCD group after 1 and 2 months of diet intervention. γ-linolenic acid (18:3n-6) was higher (P<0.05) only after 1 month and did not differ between groups after 2 months (Figures 2 to [Fig f03] [Fig f04] 5).

**Figure 5:**
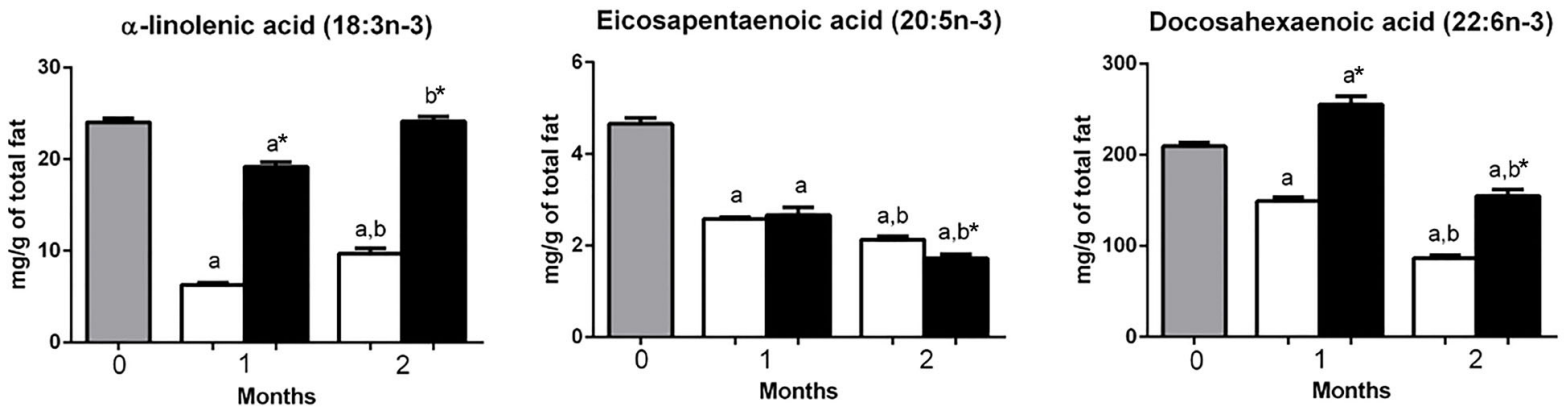
Polyunsaturated n-3 fatty acids (n-3 PUFAs) composition in the gastrocnemius muscle of mice at 0 (before starting the diets, gray bars) or fed with a high-carbohydrate diet (HCD, white bars) or high-fat diet (HFD, black bars) for 1 or 2 months. The contents of n-3 PUFAs are reported as means±SE. *P<0.05 compared to HCD group (Student's t-test). ^a^P<0.05 compared to time 0; ^b^P<0.05 compared to 1 month (one-way ANOVA).

We observed an increase (P<0.05) in myristic acid (14:0), palmitoleic acid (16:1n-7), 7-hexadecanoic acid (16:1n-9), vaccenic acid (18:1n-7), and oleic acid (18:1n-9) content (Time 0 *vs* 2 months) in both HCD and HFD groups (Figures 2 and 3).

The contents of stearic acid (18:0), heneicosanoic acid (21:0), tetracoisanoic acid (24:0), arachidonic acid (20:4n-6), docosatetraenoic acid (22:4n-6), docosapentaenoic acid (22:5n-6), eicosapentaenoic acid (20:5n-3), and docosahexanoic acid (22:6n-3) decreased in both HFD and HCD after 2 months (Figures 2, 4, and 5).

**Figure 6:**
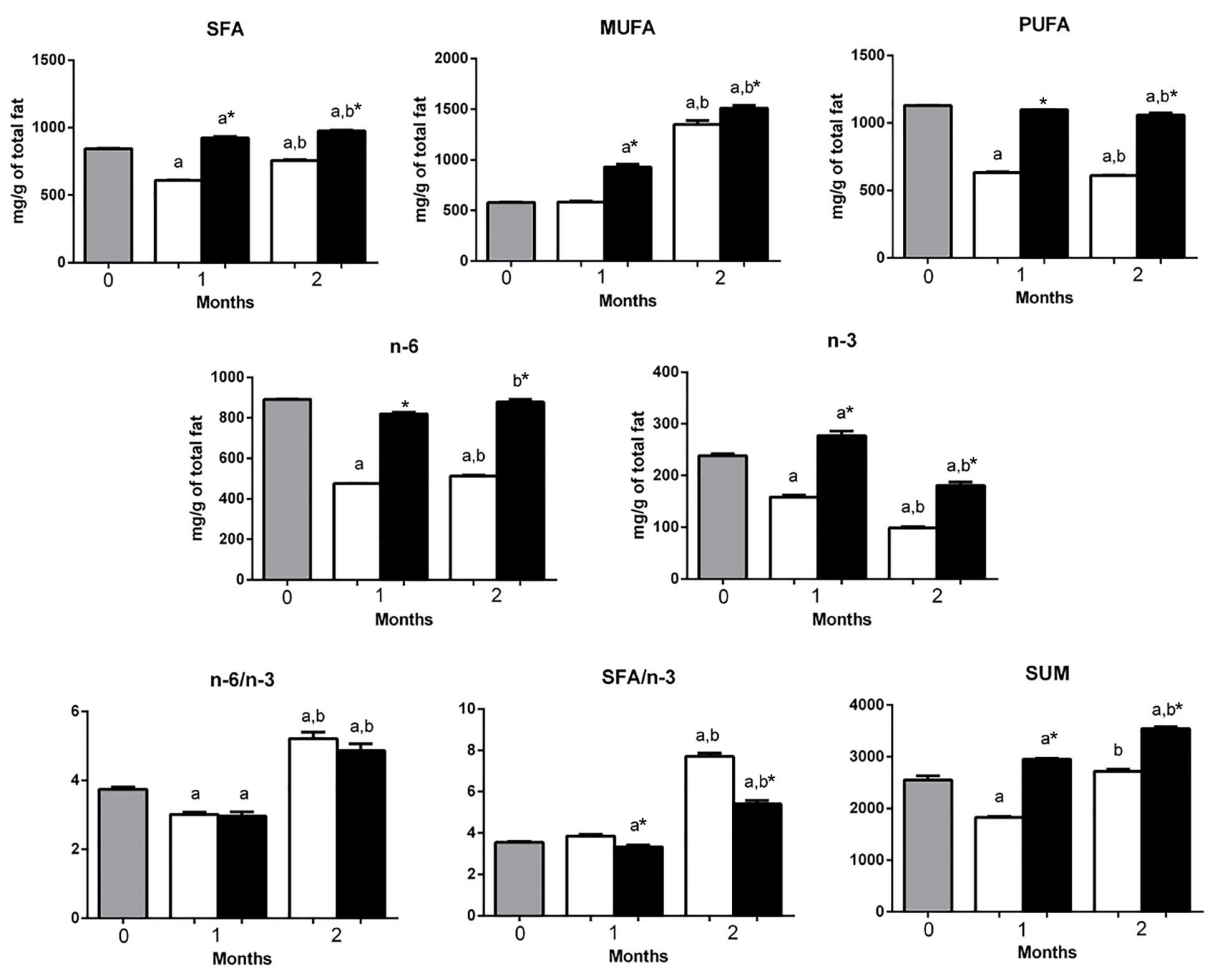
Fatty acid family composition and the n-6/n-3 and SFA/n-3 ratios in the gastrocnemius muscle of mice at 0 (before starting the diets, gray bars) or fed with a high-carbohydrate diet (HCD, white bars) or high-fat diet (HFD, black bars) for 1 or 2 months. Results are reported as means±SE. *P<0.05 compared to HCD group (Student's *t*-test). ^a^P<0.05 compared to time 0; ^b^P<0.05 compared to 1 month (one-way ANOVA). SFA: total saturated fatty acids; MUFA: total monounsaturated fatty acids; PUFA: total polyunsaturated fatty acids; SUM: sum of all fatty acids evaluated.

The HCD group had lower (P<0.05) deposition of saturated fatty acids (SFAs), monounsaturated fatty acids (MUFAs), n-6 polyunsaturated fatty acids (PUFAs), and n-3 PUFAs compared to HFD after 1 and 2 months (Figure 6).

Total fat accumulation, calculated by the sum of all FA, was more significant for the HFD group after 1 and 2 months of starting the diets (HFD *vs* HCD groups) (Figure 6).

**Figure 7:**
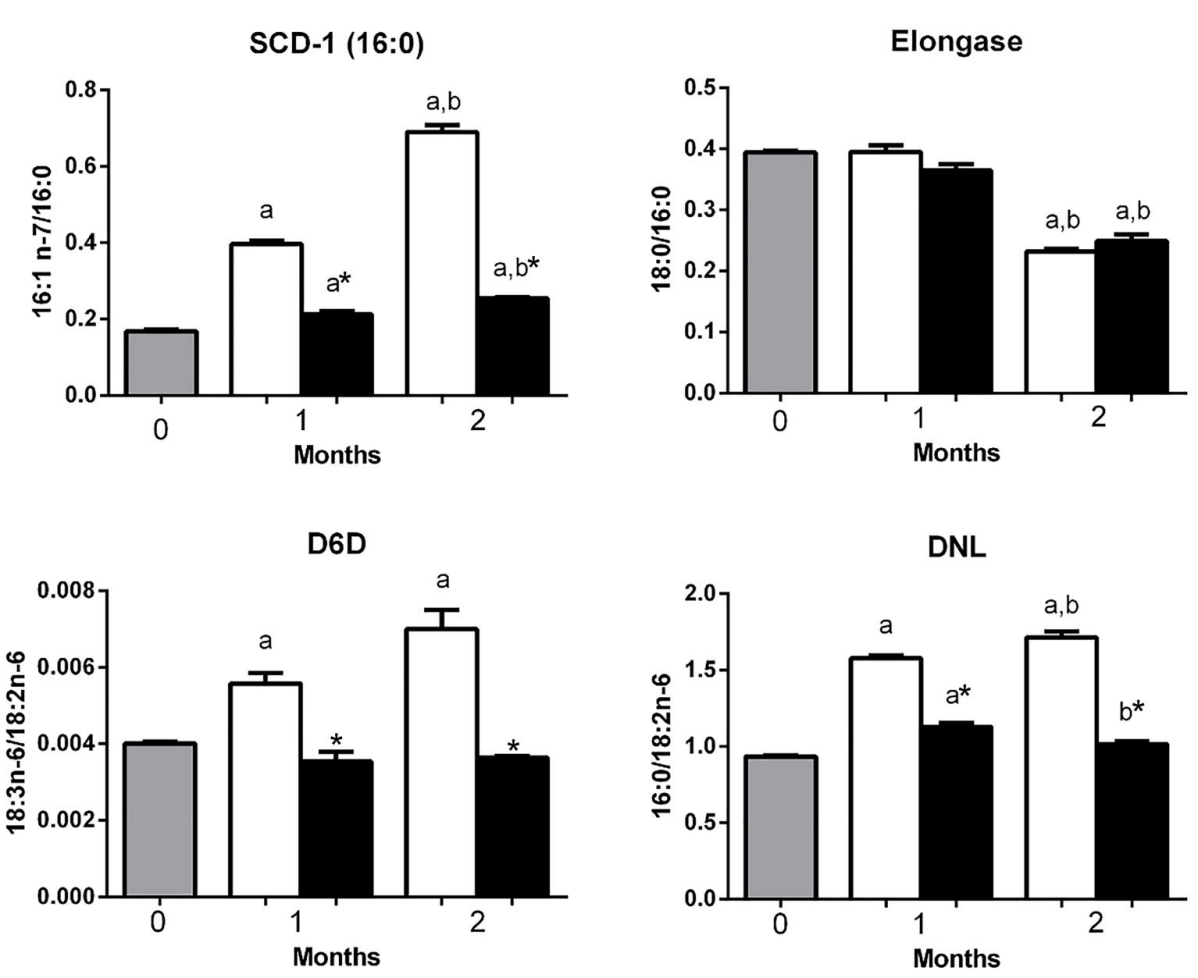
Activities of SCD-1, D6D, elongase 6, and *de novo* lipogenesis (DNL) in the gastrocnemius muscle from mice at 0 (before starting the diets, gray bars) or fed with a high-carbohydrate diet (HCD, white bars) or high-fat diet (HFD, black bars) 1 or 2 months. Results are reported as means±SE. *P<0.05 compared to HCD group (Student's *t*-test). ^a^P<0.05 compared to time 0; ^b^P<0.05 compared to 1 month (one-way ANOVA). SCD-1: stearoyl-CoA desaturase-1; Δ-6 desaturase (D6D), DNL: *de novo* lipogenesis.

The HCD group exhibited a higher (P<0.05) SFA/n-3 ratio than the HFD group 1 and 2 months after starting the diets. However, there was no difference in the n-6/n-3 ratio (Figure 6).

DNL (16:0/18:2n-6), SCD-1 (16:1n-7/16:0), and D6D (18:3n-6/18:2n-6) activities were higher (P<0.05) in HCD compared to HFD group after 1 or 2 months of diet interventions. Elongase 6 activity did not differ between groups after 1 or 2 months of diet interventions (Figure 7).

### Inflammation assessment

**Figure 8:**
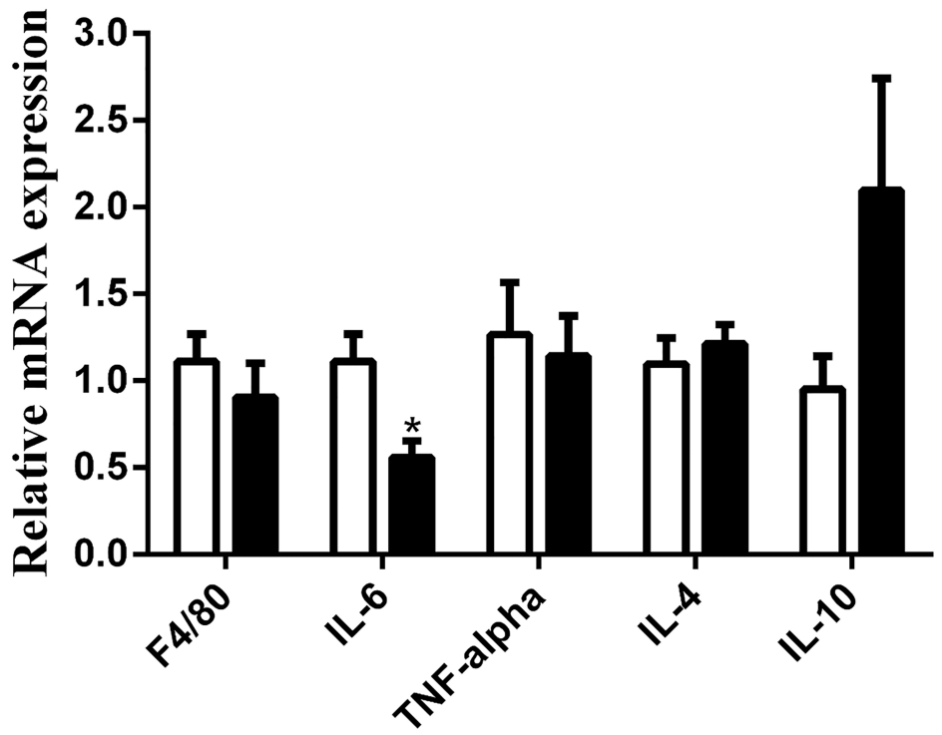
mRNA expression in gastrocnemius muscle of mice fed with either high-carbohydrate diet (HCD group, white bars) or high-fat diet (HFD group, black bars) for 2 months. β2-microglobulin (β2m) was used as the housekeeping gene. Results are reported as means±SE (n=8–10). *P<0.05 compared to the HCD group (Student's *t*-test). TNF-α: tumor necrosis factor alpha; IL: interleukin.

The HCD group exhibited higher (P<0.05) MPO activity after 2 months of diet interventions. The values (Ab 460 nm) reported as means±SE of 8–10 mice per group were: 0.40±0.02 in the HCD group and 0.33±0.01 in the HFD group. IL-6 mRNA expression was increased (P<0.05) in the HCD group compared to the HFD group after 2 months (Figure 8).

## Discussion

Macronutrient composition modulates FA deposition and inflammation in different tissues such as liver (14), brain (15), adipose tissue (20,21), and serum (17,21).

The HFD group had the same final body weight as the HCD group, despite lower caloric intake. These results are in agreement with other studies indicating that calories in the form of fat favor higher fat deposition compared with a high-carbohydrate diet (8,20).

The HFD group exhibited lower DNL activity, indicating a lower conversion of carbohydrates to lipids. However, the HFD group had a higher content of SFAs, MUFAs, PUFAs, and total lipid accumulation in the gastrocnemius muscle, which reflects at least in part the five times higher quantity of FA in this diet compared with HCD (14). Therefore, the higher DNL found in the gastrocnemius muscle of the HCD group did not compensate for the higher lipid intake of the HFD group. High fat intake may increase lipoprotein lipase activity and triacylglycerol accumulation (22).

SCD-1 activity was elevated in HCD mice, as reported by others in skeletal muscle (23). This higher activity leads to the generation of palmitoleic acid (16:1n-7), vaccenic acid (18:1n-7), and oleic acid (18:1n-9) (24).

The deposition of PUFAs strongly correlates with dietary FA availability as reported for linoleic acid (18:2n-6) and α-linolenic acid (18:3n-3) (25). This agrees with the higher quantities of linoleic acid (18:2n-6) and α-linolenic acid (18:3n-3) found in the gastrocnemius muscle of the HFD mice.

Arachidonic acid (20:4n-6) is a precursor of pro-inflammatory prostaglandins, thromboxanes, and leukotrienes (26), whereas docosahexaenoic acid (DHA, 22:6n-3) is a precursor of anti-inflammatory mediators (27). Therefore, the increased arachidonic acid (20:4n-6) and the reduced docosahexaenoic acid (DHA, 22:6n-3) contents in the HCD group after 2 months of diet interventions suggested a higher inflammatory state in the muscle of this group. In agreement with this postulation, inflammation was more significant in the HCD group, which showed higher IL-6 expression and MPO activity after 2 months of diet interventions. The skeletal muscle itself and muscle tissue infiltrating inflammatory cells produce IL-6, as observed in type 2 diabetes and inflammatory myopathies (28). MPO activity is a marker of neutrophil infiltration, which is related to cell regeneration activity after tissue injury (29). Both IL-6 expression and MPO activity are predictors of obesity-associated morbidities, pre-diabetes, and cardiovascular diseases (30).

The higher inflammation contrasted with the lower muscle FA accumulation in the HCD group. In our previous work, we found a higher accumulation of FA and inflammation intensity in the liver of the HCD group compared with the HFD group, as a consequence of more elevated DNL associated with high-carbohydrate ingestion (14).

The SFA/n-3 PUFA ratio was higher in skeletal muscle of the HCD group, whereas the n-6/n-3 PUFA ratio remained unchanged. These results are in agreement with Rasic-Milutinovic et al. (31) who reported that the SFA/n-3 PUFA ratio is a better indicator of inflammation than the n-6/n-3 PUFA ratio, considering the inflammatory properties of SFA and n-6 and the anti-inflammatory properties of n-3 PUFAs (25,27,32).

The short period (2 months) of the diet interventions was a limitation of this study. Long-term studies are necessary to clarify the development of skeletal muscle inflammation and fatty acid composition in Swiss mice fed a high-carbohydrate or a high-fat diet.

In conclusion, our results demonstrated the importance of differentiating the roles played by macronutrient composition and lipid deposition. Furthermore, contrasting with the well-established idea that increased lipid deposition is associated with more intense inflammation, the HCD was more potent to induce skeletal muscle inflammation than the HFD, regardless of the lower lipid accumulation.
